# Visible Meta‐Displays for Anti‐Counterfeiting with Printable Dielectric Metasurfaces

**DOI:** 10.1002/advs.202308687

**Published:** 2024-02-11

**Authors:** Jintao Gong, Lingxing Xiong, Mingbo Pu, Xiong Li, Xiaoliang Ma, Xiangang Luo

**Affiliations:** ^1^ National Key Laboratory of Optical Field Manipulation Science and Technology Chinese Academy of Sciences Chengdu 610209 China; ^2^ State Key Laboratory of Optical Technologies on Nano‐Fabrication and Micro‐Engineering Institute of Optics and Electronics Chinese Academy of Sciences Chengdu 610209 China; ^3^ Key Laboratory for Information Science of Electromagnetic Waves (MoE) Fudan University Shanghai 200433 China; ^4^ College of Materials Sciences and Opto‐Electronic Technology University of Chinese Academy of Sciences Beijing 100049 China

**Keywords:** high‐aspect‐ratio dielectric metasurfaces, high‐refractive‐index *TiO*_2_‐polymer composite, mass‐producible meta‐displays, polarization‐multiplexed information encoding, visible meta‐optics

## Abstract

Metasurfaces, 2D arrays of nanostructures, have gained significant attention in recent years due to their ability to manipulate light at the subwavelength scale. Their diverse applications range from advanced optical devices to sensing and imaging technologies. However, the mass production of dielectric metasurfaces with tailored properties for visible light has remained a challenge. Therefore, the demand for efficient and cost‐effective fabrication methods for metasurfaces has driven the continuing development of various techniques. In this research article, a high‐throughput production method is presented for multifunctional dielectric metasurfaces in the visible light range using one‐step high‐index *TiO*_2_‐polymer composite (TPC) printing, which is a variant of nanoprinting lithography (NIL) for the direct replication of patterned multifunctional dielectric metasurfaces using a TPC material as the printing ink. The batch fabrication of dielectric metasurfaces is demonstrated with controlled geometry and excellent optical response, enabling high‐performance light‐matter interactions for potential applications of visible meta‐displays.

## Graphical abstract

The adaptability of TPC has been demonstrated and its potential for large‐scale production of dielectric metasurfaces suitable for practical applications. The integration of the TPC printing material with the cost‐effective UV‐NIL technique positions these dielectric metasurface replicas as promising contenders in the field of meta‐display technology, with particular relevance to the upscale liquor anti‐counterfeiting industry.

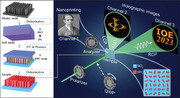

## 1 Introduction

Metasurfaces that composed of ultrathin subwavelength optical scatterer arrays represent a transformative departure from the conventional bulky and heavy optics, ushering in a new era of compact and lightweight optical devices.^[^ ^1^, ^2^, ^3^, ^4^, ^5^, ^6^ ^]^ The metasurfaces harness the powerful interaction between electromagnetic (EM) waves and optical scatterers to efficiently modulate light at the subwavelength scale, a capability distinct from traditional refractive components that operate at the wavelength scale. By configuring the optical scatterers, metasurfaces enable precise control over various aspects of EM waves, including their amplitude, phase, polarization, and frequency, opening the door to a multitude of promising applications such as beam focusing,^[^ ^7^, ^8^, ^9^, ^10^, ^11^ ^]^ holographic display,^[^ ^12^, ^13^, ^14^, ^15^, ^16^, ^17^ ^]^ cloak of invisibility,^[^ ^18^ ^]^ structural color printing,^[^ ^19^ ^]^ directional Janus functionality,^[^ ^20^ ^]^ and numerous others.^[^ ^21^, ^22^, ^23^, ^24^, ^25^ ^]^

Meta‐displays leverage metasurface technology to manipulate optical attributes, allowing for improvements in display quality, resolution, and functionality. Meta‐displays possess the potential to redefine the way we view and interact with visual content. Due to the exceptional capacity of metasurfaces to manipulate EM fields, it is possible to enable the creation of holographic and nanoprinting images with unparalleled spatial resolution. The fusion of holography and nanoprinting represents a highly efficient strategy for information multiplexing. However, existing methods primarily rely on the interleaving or stacking of nanostructures with distinct functionalities to construct multiplexed metasurfaces, effectively resulting in combinations of individual metasurfaces without increasing their information capacity. Recently, a notable trend has emerged in merging holography and nanoprinting within a single metasurface, presenting an innovative and artistic approach to information multiplexing.^[^ ^26^, ^27^, ^28^ ^]^ The essence of this approach lies in a combination between intensity modulation, following Malus's law, and phase manipulation grounded in both propagation phase and geometric phase. Within this design, three distinct images are seamlessly stored in a single metasurface. By using different incident polarizations and tailored optical setups as decoding keys, the encoded information can be faithfully reproduced in the form of a continuous grayscale image directly on the metasurface, as well as two holographic images in the far field. This innovative approach offers an efficient and crosstalk‐free solution for information multiplexing, with promising applications across diverse fields, including premium anti‐counterfeiting, high‐density optical storage, information concealment, and beyond.^[^ ^29^, ^30^, ^31^, ^32^, ^33^ ^]^ The most direct application scenario for meta‐displays lies in their use as anti‐counterfeiting inscriptions, particularly in high‐end liquor and spirits. As a matter of fact, our specially designed and manufactured three‐channel meta‐display system demonstrates remarkable effectiveness in countering counterfeiting. Initially, the near‐field structural gray‐scale nanoprinting image can be effortlessly observed using a commercially available polarizing microscope, providing the first layer of security. To enhance authentication, a laser optical path detection is implemented. Illuminating the sample with left‐circularly polarized (LCP) light reveals one far‐field holographic image visible to the naked eye on the receiving screen, establishing the second layer of the security mechanism. If additional verification is required, adjusting the incident light to right‐circularly polarized (RCP) light unveils another far‐field holographic image on the receiving screen under ambient conditions, constituting the third layer of the security mechanism. Importantly, these all‐dielectric meta‐displays were fabricated using a high‐throughput approach, signaling their potential readiness for deployment in large‐scale anti‐counterfeiting situations.

Although meta‐displays show great promise, several challenges still need to be addressed. The fabrication of complex meta‐atom patterns at large scales is a significant hurdle that researchers are actively working to overcome. Additionally, improving the efficiency and reducing the energy requirements for meta‐display systems is an open‐ended research focus. Another future directions in meta‐display include exploring new materials, such as phase change materials (PCM) and printable high‐refractive‐index materials (PHRIM), to further enhance the properties of meta‐atoms. As far as fabrication technique is concerned, to produce sub‐100 nm resolution metasurfaces, electron‐beam lithography (EBL) is commonly utilized, where a high‐voltage electron beam is directly projected onto an electron‐sensitive resist to create the desired pattern.^[^ ^34^, ^35^, ^36^ ^]^ EBL's maskless writing capability provides it with a high degree of freedom, and the short de Broglie wavelength of electrons enables the generation of high‐resolution nanostructures. Nevertheless, EBL necessitates expensive high‐vacuum systems along with a set of magnetic lenses to precisely control the rapid electrons, resulting in inherent low throughput, which hampers mass production of nanostructures. Similarly, focused ion beam (FIB) lithography is another direct‐writing method where ion beams replace electrons to erode the sample's surface with nanometer precision.^[^ ^37^ ^]^ However, FIB lithography is also unsuitable for large‐scale, high‐volume manufacturing due to its inherent drawbacks of slow speed and high cost. In the pursuit of high‐throughput metasurface fabrication, various optical nanofabrication techniques, including plasmonic lithography,^[^ ^38^, ^39^, ^40^ ^]^ interference lithography,^[^ ^41^ ^]^ and holographic lithography,^[^ ^42^ ^]^ have been explored. Unfortunately, these processes share a common limitation of the optical patterning diffraction limit, thus creating a demand for more advanced nanofabrication methods. In contrast to the aforementioned nanopatterning processes, nanoimprint lithography (NIL) relies on straightforward mechanical steps to produce nanostructures.^[^ ^43^ ^]^ Two conventional NIL methods, thermal‐NIL and UV‐NIL, are typically employed. Thermal‐NIL hardens a thermoplastic polymer resin using heat, while UV‐NIL employs UV light to solidify the resin. Consequently, UV‐NIL, with its simplified system and rapid response time, offers higher productivity, making it an attractive choice for achieving practical, high‐throughput metasurface fabrication in this study. Moreover, it should be noted that the mass production of visible meta‐displays (e.g., anti‐counterfeiting inscriptions) holds significant importance for the following several reasons. First, mass production allows for economies of scale, reducing the cost per unit of meta‐displays. This makes meta‐display technology more affordable and accessible to a wider range of consumers or industries. Second, large‐scale production enables broader market availability, meeting the demand for meta‐displays in various applications. This accessibility can drive widespread adoption of the technology across different sectors. Third, mass production encourages investment in research and development, leading to continuous improvements in meta‐display technology. The scalability of production often accelerates innovation, promoting the development of more advanced and efficient meta‐display solutions. In brief, the mass production of meta‐displays not only addresses economic considerations by reducing costs but also plays a pivotal role in advancing technology, expanding market reach, and fostering sustainability.

## 2 Results and Discussion

### 2.1 Single‐Step Printing Platform for Visible Meta‐Displays

**Figure 1:**
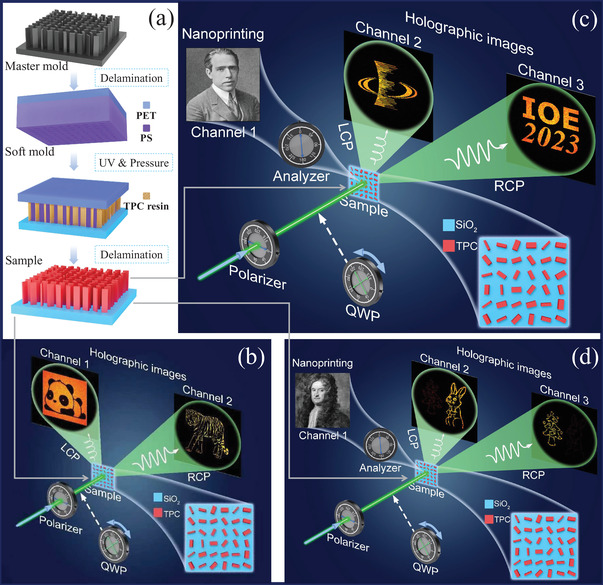
Conceptual illustration of massively produced visible meta‐displays through single‐step *TiO*_2_‐polymer composite printing. a) Fabrication flowchart of dielectric meta‐displays. b) Schematic diagram of spin‐controlled two‐channel meta‐display. c,d) Schematic diagrams of two separate three‐channel meta‐display samples for simultaneous near‐field meta‐nanoprinting and far‐field meta‐holography.

Generally, state‐of‐the‐art metasurfaces designed to function within the visible spectrum (e.g., 532 nm) are typically manufactured through complex and demanding procedures. These procedures involve depositing a transparent dielectric material like *TiO*_2_, *GaN*, or *Si*_3_ *N*_4_, followed by lithographic patterning, additional depositions, and etching processes.^[^ ^44^ ^]^ The quest for a cost‐effective alternative material platform and fabrication method, one that is accessible, scalable, and utilizes only non‐toxic disposable materials, has the potential to significantly broaden the reach of metasurface based flat optics within society. Since PHRIM usually consists of dielectric nanoparticle inclusion in a matrix of UV‐curable resin, *TiO*_2_ nanoparticles are employed to generate a *TiO*_2_‐polymer composite (TPC) for one‐step technological process. In this context, as illustrated in **Figure** **1**a, our research group shows a straightforward approach for achieving high‐throughput metasurface fabrication using a one‐step UV‐curable resin printing method. The resulting meta‐display samples, rapidly and massively replicated, demonstrate strong performance in spin‐dependent holographic imaging without/with grayscale intensity imaging, as shown in Figure 1b‐d. Moreover, we achieve large‐area, low‐cost production of these 955 nm thick metasurfaces, with feature sizes as small as 90 nm and aspect ratios reaching 11, all through a single‐step UV nanoimprint lithography process combined with a TPC printing ink (namely IOC‐133, provided by Inkron, NAGASE Group). It is worth noting that this work represents the pilot experiment of utilizing the TPC material IOC‐133 as the sole printing material for achieving high‐volume metasurface manufacturing.

While the concept of introducing nano‐particles into polymers has been recently applied in the development of large‐scale metasurface fabrication,^[^ ^45^, ^46^, ^47^ ^]^ it is important to highlight that the batch fabrication of visible meta‐displays using commercially available products remains largely unexplored. Specifically, the synthetic TPC material IOC‐133, utilized in our research, is composed of a polysiloxane matrix and *TiO*_2_ nanoparticles as inclusions, with an 80% *TiO*_2_ weight ratio. Notably, conventional TPC materials commonly employ acrylate as the matrix. In contrast to previously reported TPC, our TPC exhibits improved demolding efficiency, attributed to the distinctive properties of the polysiloxane matrix, including flexibility, water repellency, and low surface tension. Aiming to utilize our proposed TPC material IOC‐133 efficiently, we have tailored the development of an inorganic–organic hybrid polymer. This customized polymer facilitates the straightforward fabrication of transparent working stamps, offering a cost‐effective alternative to quartz stamps in UV‐NIL. The resulting working stamp, referred to as a polystamp (PS), not only boasts excellent mechanical properties but also exhibits sufficient bulk flexibility. In contrast to the frequently employed PDMS soft mold, the PS soft mold presents three distinct advantages: 1) Elevated mechanical stability: This stems from the incorporation of inorganic substance, enhancing the overall robustness of the stamp. Therefore, it can be thought to have a superior durability compared to PDMS soft mold; 2) Efficient fabrication process: The PS expedites the fabrication timeline by eliminating the necessity for baking time, contributing to a faster and more streamlined production process. It should be noted that the total baking time for PDMS soft stamp is at least 4 h;^[^ ^45^, ^46^, ^47^ ^]^ 3) Low surface tension: In our replication experiments, we do not coat any delamination agent on the PS soft mold, which clearly proves we eliminate the need for surface treatment in the detaching process. Overall, a universal framework for low cost and high volume manufacturing of high‐efficiency visible meta‐displays by simply printing high‐aspect‐ratio TPC nanostructures is proposed in this work. As a proof‐of‐concept demonstration, a batch of dielectric metasurfaces for meta‐displays in the visible spectrum is fabricated via a high‐throughput manner based on UV‐NIL. The imprinted nanostructures of three selected replicas are microscopically characterized to draw a comparison. In addition, a series of comparative analyses of the experimental measurement results for the corresponding replicas are also conducted. Noticeably, the single‐step printing platform presented here is compatible with silicon (Si) templates produced via continuously developing deep/extreme UV lithography, opening the path for inexpensive batch fabrication of consumer‐oriented multifunctional dielectric metasurfaces and offering significant prospects for the commercialization of visible meta‐displays.

### 2.2 Design of High‐Efficiency Meta‐Atoms

**Figure 2:**
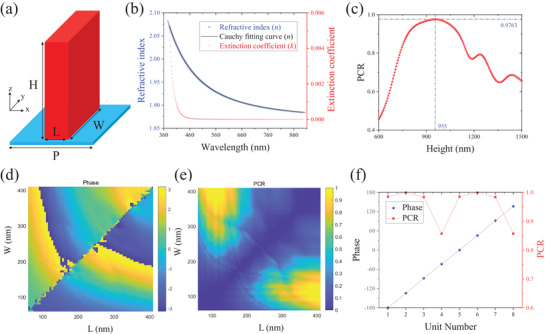
Characteristics of *TiO*_2_‐polymer composite (TPC). a) Schematic illustration of unit cell structure (i.e., meta‐atom) consisting of TPC nanofin and quartz substrate. Note: Length, indicated by L; Width, indicated by L; Height, indicated by H; Period, indicated by P. b) The measured refractive index values of TPC printing material with its corresponding fitted Cauchy dispersion curve, and the measured extinction coefficient of the TPC. c) Height optimization result of a typical meta‐atom with L = 400 nm, W = 120 nm, and P = 450 nm. d) Numerical simulation result of propagation phase delays of meta‐atoms. e) Numerical simulation result of polarization conversion ratio (PCR) of meta‐atoms. f) The propagation phase delays and PCRs of eight selected meta‐atoms.

The key to employing UV‐NIL as a single‐step fabrication method lies in the direct utilization of the printable resin as the intended meta‐atom, as depicted in **Figure** **2**a. Typically, resins utilized in UV‐NIL exhibit a refractive index (*n*) range of approximately 1.5–1.6, thus making them unsuitable for use as meta‐atoms in optical metasurfaces due to the requirement of extremely large structural parameters. To enhance the refractive index (*n*) of the resin within the visible spectrum, a well‐established and cost‐effective approach involving the synthesis of *TiO*_2_ nanoparticles is adopted to create a TPC. This is achieved by dispersing *TiO*_2_ nanoparticles with diameters smaller than 20 nm into a UV‐curable resin, which serves as a matrix for facilitating the direct transfer of nanostructures from the master mold. The synthetic TPC is a homogeneous effective medium with an elevated effective refractive index, more closely resembling that of *TiO*_2_ than the pristine resin. To ascertain the refractive index (*n*) and extinction coefficient (*k*) of TPC, a 400 nm thick TPC thin film is spin‐coated onto a Si substrate. Next, the amplitude ratio (Ψ) and phase difference (Δ) between the *s* and *p* components of this thin film are determined through ellipsometry. Finally, the obtained Ψ and Δ data are fitted using the Cauchy dispersion model, which is represented by the following equation:

(1) $$n\left(\lambda \right)=n_{0}+\frac{n_{1}}{\lambda^{2}}+\frac{n_{2}}{\lambda^{4}}$$

where *n* is the refractive index, λ denotes the wavelength in the unit of (*nm*), and *n*_0_, *n*_1_, *n*_2_ are coefficients to match the Cauchy model using the measured data, as shown in Figure 2b. From Figure 2b, it can be easily found that Cauchy fitting curve features *n*_0_ = 1.867, *n*_1_ = 1.059 × 10^4^ (*nm* ^2^), and *n*_2_ = 2.800 × 10^9^ (*nm* ^4^) for TPC printable resin used in this work. Efficiently manipulating the EM wavefront has long been a primary goal in the field of geometric metasurfaces. A design strategy aimed at achieving high optical efficiency is highly sought after. Since geometric phase arises from photonic spin‐orbit interaction (PSOI), an additional phase delay is introduced only to the cross‐polarization component when circularly polarized (CP) light is incident. Consequently, the pursuit of high‐efficiency geometric metasurfaces hinges on achieving significant polarization conversion ratio (PCR) between the two opposing circular polarization (CP) states. Considering the height/thickness of meta‐atoms can significantly impact the PCR of designed meta‐displays. Therefore, a typical meta‐atom with length L = 400 nm, width W = 120 nm, and period P = 450 nm is initially chosen to perform a height optimization for maximizing the PCR of designed meta‐displays. The commercial software Lumerical FDTD (Finite‐Difference Time‐Domain) is used to conduct height scanning of the meta‐atom (full‐wave simulation), the corresponding height optimization result shown in Figure 2c clearly exhibits that the meta‐atom height features an optimized value of H = 955 nm. Moreover, with the optimized height H = 955 nm and a fixed period P = 450 nm, we conducted a phase mapping and a PCR mapping of the meta‐atoms by sweeping lengths (L) from 70 to 410 nm and widths (W) from 70 to 410 nm, the corresponding results are shown in Figure 2d,e, respectively. Next, we introduce propagation phase to effectively divide the phase modulation channel into two distinct and independent channels. As illustrated in Figure 2f, through meticulous selection of the nanostructure dimensions based on Figure 2d,e, we can attain an eight‐step propagation phase (for more details, refer to Table S2, Supporting Information) with exceptional performance (i.e., uniformly distributed eight‐step phase responses and an ultrahigh average PCR of 95.6%). The low anisotropy observed in Unit #4 and Unit #8 (as detailed in Table S2, Supporting Information), both nearing square shapes, results in a reduced PCR. Fortunately, in the context of optical metasurface holography, which is characterized by its collective global outcomes, the effects of local inconsistencies or anomalies are minimal. Therefore, calculating an averaged PCR value across these eight unit structures is a reasonable approach. Furthermore, since these eight nanostructures (i.e., meta‐atoms) maintain their role as half‐waveplates as per our selection, we can combine the geometric and propagation phases to create distinct phase delays for LCP and RCP incident light, thus enabling the establishment of two entirely separate information channels for phase modulation.

### 2.3 Principle of Polarization‐Multiplexed Visible Meta‐Displays

The design principle of spin‐decoupled two‐channel meta‐display leverages a combination of propagation and geometric phase techniques to embed dual phases. To reconstruct separate hologram wavefronts for RCP and LCP light incidence, the target phase maps denoted by Φ_*RCP*_ and Φ_*LCP*_, respectively, can be written as follows:

(2) $$\Phi_{RCP}=\varphi +2\sigma_{+}\theta$$

(3) $$\Phi_{LCP}=\varphi +2\sigma_{-}\theta$$

where φ signifies the propagation phase of meta‐atom, σ_±_ indicates the spin of CP light (σ_+_ = 1 denotes RCP, σ_−_ = −1 denotes LCP), and θ represents the orientation angle of meta‐atom used for geometric phase modulation. Therefore, the selection and the orientation/rotation angle of meta‐atoms into the two‐channel meta‐display can be determined through the following mathematical formulations:

(4) $$\varphi =\frac{\Phi_{RCP}+\Phi_{LCP}}{2}$$

(5) $$\theta =\frac{\Phi_{RCP}-\Phi_{LCP}}{4}$$

Equations (4) and (5) delineate the conditions necessary for breaking symmetry, which are essential to achieve asymmetric transmission with chiral nanostructures when exposed to RCP and LCP light. These two equations guide the configuration of the phase mask, determining its necessary dimensions and rotation angles of meta‐atoms within the dielectric dual‐channel meta‐display.

Furthermore, the methodology applied in this work also allows for the integration of three entirely distinct images (a near‐field nanoprinting image and two far‐field holographic images) into a single meta‐display. Specifically, a continuous grayscale nanoprinting image becomes visible on the sample surface when placed within an orthogonal‐polarization optical pathway. Concurrently, two separate holographic images are projected into the far field when the multistep phase‐modulated meta‐display is illuminated with LCP and RCP laser light, respectively. To realize the three aforementioned independent information channels within a meta‐display, we use nanofins that function as nano‐half‐waveplates. These encode three distinct channels under varying polarization controls. Initially, the amplitude modulation is considered for capturing the nanoprinting image. It is recognized that a half‐waveplate can alter the polarization direction of incident linearly polarized (LP) light. Thus, a meta‐display made of nano‐half‐waveplates can modulate the amplitude (or intensity) of incoming light at individual pixel levels, forming a grayscale image solely by adjusting the orientations of these nano‐half‐waveplates. As depicted in Figure 1c,d, when the meta‐display is situated within an orthogonal‐polarization optical pathway, the resultant output light intensity, designated by *I*, can be represented as:

(6) $$I=I_{0}\sin^{2}\left(2\theta \right)$$

where *I*_0_ signifies the incident light intensity and θ denotes the orientation angle of each meta‐atom (i.e., nano‐half‐waveplate). According to Equation (6), it can be comprehensively understood that four orientation angles (i.e., θ, $\frac{\pi }{2}-\theta$, $\frac{\pi }{2}+\theta$, π − θ) providing different geometric phases for phase manipulation can generate an equal output light intensity due to the orientation degeneracy based on Malus's law. This intriguing fourfold degeneracy relation between intensity and orientation angle implies a new degree‐of‐freedom for controlling the geometric phase of incoming light on a given meta‐display. Furthermore, by integrating the aforementioned phase modulation with Malus's law based amplitude modulation, we can encode three distinct images onto a single meta‐display, which results in one near‐field nanoprinting image and two far‐field holographic images. This foundational approach paves the way for a polarization‐dependent three‐channel meta‐display tailored for information multiplexing.

### 2.4 Microscopical Characterisation of Massively Fabricated Replicas

The direct NIL printing process involves a replication technique where a home‐made PS soft mold reproduces the complementary patterns from a Si master mold. These patterns are then reversed into the printing material TPC to recreate the original nanostructures within the Si master mold. The Si master mold consisting of 955 nm tall nanofins is patterned via EBL and then etched into the Si substrate through reactive ion etching. The meta‐displays made of TPC are designed for a wavelength of 532 nm, with a minimum feature size (i.e., critical dimension, CD) of 90 nm, a maximum aspect ratio of around 11, and a refractive index of 1.94 (λ = 532 *nm*) for the meta‐atoms (i.e., building blocks). The fabrication workflow (see Figure S1, Supporting Information) offers a swift and high‐resolution approach for patterning high‐aspect‐ratio dielectric nanostructures, all achieved through a single process that demonstrates strong compatibility with substrates. Once the Si master mold is prepared, a sole UV‐NIL step suffices to produce the patterned dielectric TPC on the substrate, eliminating the need for any subsequent procedures. UV‐NIL, being a rapid process, is complemented by the reusability of the soft mold, rendering the patterned dielectric TPC nanostructures highly competitive when compared to commercially produced plastic meta‐devices manufactured via injection molding. The UV‐cured TPC, attributed to its remarkable stiffness, enables the successful transfer of patterns with aspect ratios exceeding 10:1, a feat that is recognized as highly challenging with conventional sol–gel methods. The resulting TPC patterns exhibit an insignificant shrinkage (uniform and less than 3%) along each dimension, irrespective of the number of reproductions/replicas.

**Figure 3:**
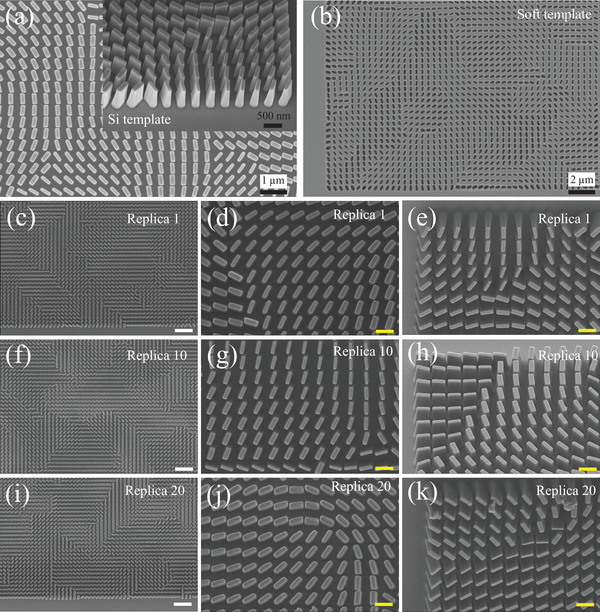
Scanning electron microscopy (SEM) characterization of massively fabricated replicas. a) SEM image of Si master template in the top view. Inset: Close‐up SEM image of the Si master template in the perspective view. b) SEM image of the transitional soft template. c–e) SEM image of *TiO*_2_‐polymer composite (TPC) replica 1 in the top view, high‐magnification SEM image of TPC replica 1 in the top view, and high‐magnification SEM image of TPC replica 1 in the perspective view. f–h) SEM image of TPC replica 10 in the top view, high‐magnification SEM image of TPC replica 10 in the top view, and high‐magnification SEM image of TPC replica 10 in the perspective view. i–k) SEM image of TPC replica 20 in the top view, high‐magnification SEM image of TPC replica 20 in the top view, and high‐magnification SEM image of TPC replica 20 in the perspective view. Note: The white scale bars indicate a length of 2μm, and the yellow scale bars denote a length of 500 *nm*.

A durability experiment is carried out to assess the longevity/reusability of the stamp, involving multiple rounds of imprinting. As shown in **Figure** **3**, scanning electron microscope (SEM) images of the Si master mold, the self‐developed PS soft mold, and the 1^*st*^, 10^*th*^, and 20^*th*^ imprinted replicas (Note: The time for fabricating these 20 replicas is less than 30 min), are displayed. Upon close examination, it becomes evident that nearly every individual nanofins remains intact, as indicated by consistently bright sidewalls, a common characteristic in SEM images of vertical features. The absence of any broken features after an extended series of imprinting iterations serves as a testament to the mechanical strength of the printing TPC material after being cured. Furthermore, Figure 3c–k confirm that the imprinted features exhibit minimal distortion, and gaps as narrow as 33 nm between the nanofins have been successfully achieved. Measurements from the cross‐sectional SEM images reveal aspect ratios of up to 10.5 for the imprinted nanofins. Although it is possible that a greater number of imprints/replicas per stamp could be achieved, this study serves to illustrate that feature fidelity and stamp performance can withstand at least 20 imprints without noticeable deterioration.

### 2.5 Experimental Demonstration

**Figure 4:**
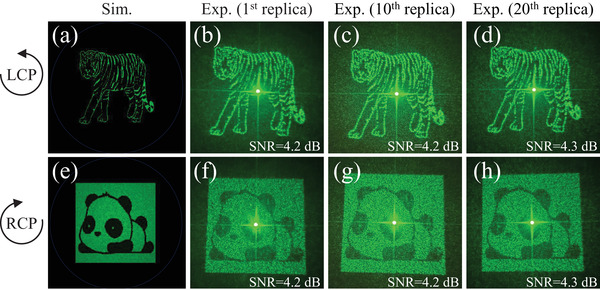
Results of two‐channel meta‐displays. a) Simulated far‐field holographic image under LCP light incidence. b–d) Experimentally observed far‐field holographic images of the 1^*st*^, the 10^*th*^, and the 20^*th*^ replicas, respectively, under LCP light incidence. e) Simulated far‐field holographic image under RCP light incidence. f–h) Experimentally observed far‐field holographic images of the 1^*st*^, the 10^*th*^, and the 20^*th*^ replicas, respectively, under RCP light incidence.

To demonstrate the versatility of spin‐selective meta‐holograms and the feasibility of high‐throughput fabrication of visible meta‐displays, we undertake the design, fabrication, and evaluation of a metasurface capable of encoding distinct holograms for RCP and LCP light. We harness iterative phase retrieval in the basis of Gerchberg–Saxton (GS) algorithm to compute near‐field phase profiles that, when imposed by a metasurface consisting of non‐interacting meta‐atoms, would yield far‐field intensity images of a cartoon Tiger and a cartoon Panda for each circular polarization in transmission. The resulting meta‐display replicas are crafted using our self‐developed TPC printing process on quartz substrate. The meta‐display sample 1 (MDS1) is designed for operation in the visible spectrum at λ = 532 nm with an area of $540\times 540\;\mu m^{2}$. Remarkably, the measured far‐field intensity profiles of replica 1, replica 10, and replica 20, upon illumination with each circular polarization, closely matched the numerical simulated images (calculated by vector diffraction theory with MATLAB), offering significant details of target images, as displayed in **Figure** **4**. The minor variations between the simulated images and the measured holograms can be attributed to fabrication imperfections and an assumption made by the phase reconstruction algorithm regarding uniform amplitude transmission at each point (x, y). It is also noteworthy that, through the methodology presented above, the phase profiles imparted on each circular polarization and the final resulting far fields can be fully decoupled. The measured hologram efficiency, defined as the ratio of the transmitted holographic image's power (i.e., power of the cross‐polarized light) to the incident CP beam's power,^[^ ^47^ ^]^ has an average value of 89.4% for all three MDS1 replicas (replica 1, replica 10, and replica 20). For more details on PCR measurements, refer to Section SI‐8 (Supporting Information). Considering that the used meta‐atoms have an average simulated efficiency of 95.6% based on PCR calculation as mentioned in Section 2.2. It is worth noting that the experimentally measured efficiency and theoretical efficiency closely align.

To showcase the batch fabrication of three‐channel meta‐displays, we design and simulate a second meta‐display sample 2 (MDS2) with an area of $540\times 540\;\mu m^{2}$. Initially, we select a high‐resolution IOE (Institutes of Optics and Electronics) logo and a character string of “IOE 2023” and numerically calculate their phase profiles for 1200 × 1200 unit elements (pixel size: 450 × 450 *nm* ^2^) in the far‐field using a modified GS algorithm. Subsequently, these calculated phase maps undergo processing through Equations (4) and (5) to generate a multiplexed phase profile. Concurrently, we compute the amplitude profile for the gray‐scale physicist Niels Bohr portrait, featuring the same area of $540\times 540\;\mu m^{2}$, with the same pixel size of 450 × 450 *nm* ^2^. In the second step, this calculated amplitude profile is discretized using Equation (6) into four different orientation options (θ, $\frac{\pi }{2}-\theta$, $\frac{\pi }{2}+\theta$, π − θ) to account for Malus's law fourfold degeneracy. While designing the MDS2, the final orientation angles of the meta‐atoms are determined by rounding the calculated geometric phase profile to the closest available options (2θ, π − 2θ, π + 2θ, 2π − 2θ). This process allows modulation of both intensity and phase profiles with one near‐field nanoprinting image and two far‐field holographic images on a single meta‐display.

**Figure 5:**
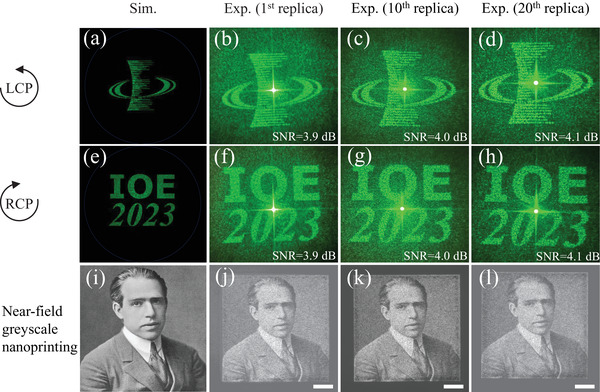
Results of three‐channel meta‐displays. a) Simulated far‐field holographic image under LCP light incidence. b–d) Experimentally observed far‐field holographic images of the 1^*st*^, the 10^*th*^, and the 20^*th*^ replicas, respectively, under LCP light incidence. e) Simulated far‐field holographic image under RCP light incidence. f–h) Experimentally observed far‐field holographic images of the 1^*st*^, the 10^*th*^, and the 20^*th*^ replicas, respectively, under RCP light incidence. i) Simulated near‐field greyscale nanoprinting image when inserted in an orthogonal‐polarization optical pathway. j–l) Experimentally observed greyscale nanoprinting images of the 1^*st*^, the 10^*th*^, and the 20^*th*^ replicas, respectively, when inserted in an orthogonal‐polarization optical pathway (all white scale bars: 100μm).

To characterize the MDS2, we initiate the assessment process by capturing the nanoprinting image of the 1^*st*^ replica of MDS2, as depicted in **Figure** **5**j, employing an optical polarizing microscope (Caikon XP‐550c). Utilizing a 10× objective and a micro digital camera (Model: CKC2000), we record the light intensity distribution in the vicinity of the MDS2 surface in transmission mode. The outcome reveals a clearly distinguishable image of the Niels Bohr picture, affirming the effective performance of the MDS2 in amplitude modulation within the near field. The appearance of minor noise speckles can be ascribed to the near‐field interaction between neighboring nanofins, a concern that can be mitigated through the implementation of a complex super‐cell design. Moreover, both the 10^*th*^ replica and the 20^*th*^ replica present closely resembling near‐field nanoprinting imaging behavior, as displayed in Figure 5k,l. Notably, the experimental observations of all three replicas (replica 1, replica 10, and replica 20) are consistent with the simulation result of near‐field greyscale nanoprinting, as shown in Figure 5i. Subsequently, we employ an alternative optical system for the observation of the resultant far‐field holographic images of the above‐mentioned three replicas (replica 1, replica 10, and replica 20), as exhibited in Figure 5b–d, f–h. The MDS2 is subjected to illumination from a tunable super‐continuum laser source (NKT‐SuperK EXTREME) operating at a specific wavelength of 532 nm. To manipulate the polarization state of the incident beam into circular polarization, a polarizer and a quarter waveplate (QWP) are employed. By rotating the QWP by 90°, we could alternate between two independent information channels, resulting in the appearance of two distinct far‐field holographic images with different configurations of the QWP. As demonstrated in Figure 5b, f, we could clearly discern either the IOE logo (LCP target image) or the character string “IOE 2023” (RCP target image) with a high degree of fidelity through polarization control from the 1^*st*^ replica of MDS2. Similarly, both the 10^*th*^ replica and the 20^*th*^ replica exhibit nearly identical far‐field holographic imaging performance, as illustrated in Figure 5c,g,d, h, respectively. Because the holographic images generated are visibly clear to the naked eye and expanded in size as the observation distance increased (i.e., the holographic images are diverging), we employ a white paper as a screen positioned 30 cm away from the sample, and then take photographs using an ordinary smartphone commonly used in daily life. Significantly, the measured LCP/RCP holograms exhibit strong agreement with the simulated LCP/RCP holographic images, as depicted in Figure 5a,e, respectively. Generally speaking, minimal cross‐talk noise is present in each channel, confirming the independence of the two information channels, enabling the realization of a three‐channel meta‐display with TPC meta‐atom design. The massively fabricated three‐channel meta‐displays with simple TPC nanostructure design, integrating holography and nanoprinting, offers a technical advantage over previous fabrication approaches. Additionally, the recorded information in the three channels requires decoding using different polarized light and optical setups, which holds promise for applications in anti‐counterfeiting. Specifically, the nanoprinting image is decoded in an orthogonal‐polarization optical path and observed through an optical microscope. Meanwhile, the holographic images are decoded using two distinct CP light sources and are observed in the far field by the naked eye or a commercial camera or an ordinary smartphone. For anti‐counterfeiting applications, the two holographic images can be designed to overlap, concealing the real information. Consequently, our fabrication approach presents a simple and versatile platform to rapidly produce multifunctional meta‐displays.

Furthermore, another three‐channel meta‐display sample 3 (MDS3) is designed and fabricated simultaneously. The massively produced replicas (in this work: replica 1, replica 10, and replica 20) of the MDS3 presented barely distinguishable near‐field nanoprinting images and almost equivalent far‐field holographic images (refer to Figure S2, Supporting Information). Noticeably, the near‐field nanoprinting images of the Isaac Newton portrait on the MDS3 replicas exhibited a nearly equivalent gray‐scale imaging performance to those of the Niels Bohr portrait on the MDS2 replicas. On the other hand, due to the spatial separation of the designed holographic images and the limited choice of eight unit structures (i.e., meta‐atoms) for independent control of LCP and RCP light incidence, it is observable in the experiment that when LCP light is incident, the produced holographic image (target pattern: a sketch of Christmas Tree) is accompanied by slight noise from the RCP generated holographic image (target pattern: a sketch of Peter Rabbit), and vice versa. The spatial separation of the holographic images (LCP pattern takes half space, while RCP pattern takes the other half) implies the absence of half of the meta‐atoms for phase control, and the ineffective half of the unit structures is destined to introduce undesired noise. It should be noted that the observed experimental results match the simulation outcomes based on vector diffraction theory since the accompanying holographic pattern (i.e., noise) originated from the opposite CP can be theoretically predicted.

**Figure 6:**
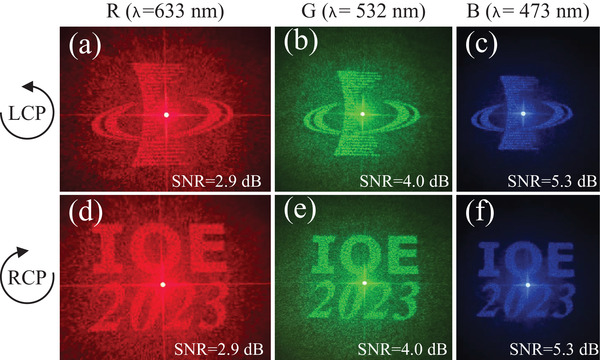
Broadband response characterisation results of the 10^*th*^ replica from the MDS2. a–c) Experimentally observed far‐field holographic images the 10^*th*^ replica using LCP light at three distinct wavelengths: 633, 532, and 473 nm, respectively. d–f) Experimentally observed far‐field holographic images of the the 10^*th*^ replica using LCP light at three distinct wavelengths: 633, 532, and 473 nm, respectively.

**Figure 7:**
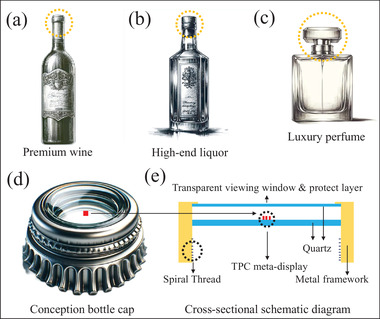
Straightforward real‐life applications of the printable meta‐display devices in high‐value product anti‐counterfeiting market. a–c) Sketch drawings of premium wine, high‐end liquor, and luxury perfume, respectively. Dashed orange circles are used to emphasize the bottle caps for incorporating meta‐display devices. d) Perspective view of a new production conception bottle cap. e) Schematic diagram of the cross‐sectional details of the newly conceptualized bottle cap.

Finally, to thoroughly evaluate the broadband response of the MDS2 replicas in the far field, we employ the above‐mentioned tunable super‐continuum laser source. This source illuminates the sample at operating wavelengths of 473 nm (blue color), 532 nm (green color), and 633 nm (red color), respectively. The experimental results of the 10^*th*^ replica of MDS2, as depicted in **Figure** **6**, showcase holographic images of high quality across the visible spectrum. For further details regarding the mechanism of the broadband response, refer to Figure S4 and Table S1‐3 (Supporting Information). On the one hand, the meta‐displays' inherent broadband spectral property enhances its suitability for practical applications by simplifying observation conditions. On the other hand, it is also important to realize that this broadband response feature makes the dual‐channel/three‐channel meta‐displays particularly robust in the face of high‐throughput fabrication and duplication errors. In fact, the primary innovation of this work lies in the development of large‐scale anti‐counterfeiting applications for real‐world scenarios, a focus that also represents our ongoing commitment. To clearly highlight this novelty, it is essential to provide a specific demonstration of how this technology can be employed in high‐value product anti‐counterfeiting. For a more comprehensive understanding, we have designed a conceptual glass bottle cap (see **Figure** **7**d for a perspective view) that can be universally applied to various expensive items, such as premium wines, high‐end liquors, and luxury perfumes (see Figure 7a–c for a visual perception). Our meta‐display devices are perfectly compatible with this anti‐counterfeiting bottle cap. From Figure 7e, it can be clearly seen that the printable anti‐counterfeiting meta‐display system is seamlessly integrated into the metal (or glass) framework of bottle cap. Moreover, a transparent observation glass window, serving also as a protective layer, is situated atop the cap. Initially, the tri‐channel meta‐display system utilizes a near‐field structural gray‐scale nanoprinting image, exemplified by a Bohr portrait, which can be easily and clearly viewed using a standard polarizing microscope, establishing the first level of security. To further enhance authentication, we can implement detection within the laser optical path. Utilizing LCP light to illuminate the sample, the IOE logo becomes visible to the naked eye on the receiving screen, whether it be white paper, a white wall, or other flat surfaces of light color in the ambient environment. This establishes the secondary layer of the security mechanism. Should additional verification be necessary, adjusting the incident light to RCP light reveals the characters“IOE 2023” visible to the naked eye on the receiving screen, such as white paper, a white wall, or other light‐colored flat surfaces, thus forming the third layer of the security mechanism. Consequently, the proposed printable tri‐channel meta‐display system is ideally suited for the high‐end product anti‐counterfeiting market. This suitability stems from its advanced triple‐layered security approach, combined with the benefits of low‐cost and high‐throughput fabrication method.

### 2.6 Discussion on Holographic Displays

In this research, our focus is on micro‐scale holographic displays. We use CP light with a beam spot size of ≈3 mm as input, without imposing any restrictions like pinhole limitations. Additionally, we do not employ a co‐polarization analyzer to filter the output holographic images. The purpose of this measurement method is to illustrate the simplicity and effectiveness of setting up holographic imaging, highlighting its practicality in real‐life scenarios. This approach, with its ease of characterization, holds considerable promise for the development of future integrated systems and swift display validation. Because we directly perform holographic display, our holographic signal is mixed with a large amount of background noise (i.e., unmodulated light). Indeed, the elimination of image noise and fluctuations is achievable. As illustrated in Figure S7 (Supporting Information), the effective approach involves filtering the unmodulated co‐polarization light, thereby obtaining a distinct holographic image. In essence, the feasibility depends on the specific application scenarios. For a rapid anti‐counterfeiting meta‐display, employing either LCP or RCP incidence allows for the immediate capture of the holographic image using a screen positioned behind the sample. Conversely, for a focus on a clear meta‐display, the implementation of an optical system (QWP + LP) becomes necessary to filter out the co‐polarization component. To quantitatively assess image clarity and evaluate the contrast of holographic signal against background noise, the Signal‐to‐Noise Ratio (SNR) of the image is introduced. The SNR of an image is defined by $SNR=10\cdot \log_{10}\frac{S}{N}$ (dB), where *S* represents the average value of the holographic signal and *N* signifies the mean value of background noise. It is worth noting that the associated SNR values are appended to each of the measured holographic images, offering a metric to gauge the quality of the holographic display. However, it is also important to acknowledge that variations in SNR values may occur due to the non‐identical conditions during holographic image capture. Factors like the size of the capture range and photography angles can contribute to noticeable fluctuations in SNR values (Note: While efforts were devoted to maintain consistent shooting conditions, some deviations are inevitable. Moreover, each change of the sample and testing at different positions introduces more challenges to achieving precise holographic image capture).

## 3 Conclusion

In summary, meta‐display represents a groundbreaking advancement in near‐field nanoprinting and far‐field holographic imaging, made possible by the unique properties of metasurfaces. By combining the principles of metasurfaces and holography, this technology offers unprecedented control over light and opens up a world of possibilities in displays, data storage, and anti‐counterfeiting. As research and development continue to push the boundaries of meta‐display, we can anticipate transformative applications that will reshape the way we perceive and interact with visual information. In this article, we have achieved a batch fabrication of dielectric metasurfaces for visible meta‐displays via one‐step TiO_2_‐polymer composite printing, featuring an ultrahigh theoretical PCR (efficiency at the meta‐atom level) of 95.6% and a consistent averaged experimental PCR (efficiency at the meta‐display level) of 89.4% at a wavelength of 532 nm. Our innovative method centers on the exclusive use of TPC as the printing material. This TPC material, boasting a high refractive index, empowers its direct application as a meta‐atom for dielectric metasurfaces that operate within the visible spectrum. Notably, for our proposed TPC material, we have specifically developed PS soft templates. The use of these PS soft templates has successfully eradicated the need for surface treatment during the detachment process. Moreover, we extend this TPC printing process to achieve both mechanical and chemical versatility, facilitating the uniform and high‐throughput fabrication of meta‐displays with a critical resolution of 90 nm (gap as small as 33 nm) and an aspect ratio exceeding ten. Furthermore, we also unveil the broadband capabilities of the TPC based replicas of designed meta‐displays by characterising holographic images with different visible wavelength inputs (Red: 633 nm, Green: 532 nm, Blue: 473 nm).

Essentially, we have demonstrated the adaptability of TPC and its potential for large‐scale production of dielectric metasurfaces suitable for practical applications. This achievement involves transferring high‐aspect‐ratio dielectric metasurfaces onto quartz substrates, paving the way for the development of multifunctional meta‐displays. The integration of the TPC printing material with the cost‐effective UV‐NIL technique positions these metasurface replicas as promising contenders in the field of meta‐display technology, with particular relevance to the upscale liquor anti‐counterfeiting industry. Although there is a constraint in the meta‐display area due to limitations in Si master mold fabrication, this restriction can be effectively addressed through EBL, even though it may require a time investment, considering that the time cost is incurred only once. Therefore, we maintain confidence that our endeavors could serve as a foundation for scaling up and achieving consistent production of meta‐displays, ultimately contributing to their successful commercialization.

## 4 Experimental Section

### Simulation

The Finite‐Difference Time‐Domain (FDTD) method in Lumerical FDTD software was used to simulate the unit cell structure and conduct height optimization. Unit cell boundaries were adopted in the x and y directions for TPC meta‐atoms, and open boundaries were adopted in the z direction for boundary conditions. To simulate the far‐field holographic images and near‐field greyscale nanoprinting images of meta‐displays, the MATLAB software was employed to perform the corresponding numerical simulations using vector diffraction theory. Furthermore, refer to Section SI‐6 (Supporting Information) for additional details regarding the unit cell structure, which features a 3D trapezoidal shape instead of the conventional 3D rectangular shape.

### Fabrication

The entire fabrication process was divided into three steps: Fabrication of silicon (Si) master mold (i.e., the 1^*st*^‐generation template), duplication of the polystamp (PS) soft mold (the 2^*nd*^‐generation template), and replication of the final meta‐display products (see Figure S5, Supporting Information). The Si master mold fabrication process for the meta‐display replicas involved the use of electron beam writing (Elionix F125) to create an identical pattern to that of the target TPC meta‐display. First, a layer of photoresist (AR‐6200) was applied to the silicon wafer, and an opposite pattern to the desired pattern was transferred to the photoresist through electron beam exposure. Second, a 50 nm thick chromium (Cr) layer was deposited onto the sample via electron beam evaporation system (ULVAC ei‐5z). Third, a lift‐off process was conducted in acetone to define a Cr hard mask on the sample. Fourth, the sample was etched along the Cr mask using inductively‐coupled plasma reactive ion etching (LEUVEN INSTRUMENTS). Fifth, the Si master mold was completed after removal of a Cr hard mask residue using Cr etchant (nitrate wet etching). Next, liquid photosensitive PS resin was coated onto the Si master mold, and a PET substrate was placed on top of the coated PS film. The synthetic soft mold (PET substrate + PS structures) was then solidified under ultraviolet light irradiation with a contact pressure of 5000 Pa. After that, the soft mold and the Si template were vertically delaminated at a slow separate speed. Finally, high‐throughput UV‐NIL fabrication process of TPC based meta‐display replicas was automatically completed via a commercial NIL equipment (GL8/12 CLIV Gen2, GermanLitho GmbH).

### Measurement

Refer to Figure S3 (Supporting Information) for a detailed description of the optical measurement setups for multi‐channel meta‐displays. For more information on the optical measurement setups of filtered multi‐channel meta‐displays, see Section SI‐7 (Supporting Information). Additionally, Section SI‐8 (Supporting Information) provides further details on the measurements of PCR efficiency.

### Statistical Analysis

The raw data was utilized to prepare graphs using Origin 2019b.

## Conflict of Interest

The authors declare no conflict of interest.

## Supporting information

Supporting Information

## Data Availability

The data that support the findings of this study are available from the corresponding author upon reasonable request.
