# Supplementary material for: Visible Meta‐Displays for Anti‐Counterfeiting with Printable Dielectric Metasurfaces
Source: Adv Sci (Weinh). 2024 Feb 11;11(17):2308687. doi: 10.1002/advs.202308687 (PMC11077653; doi:10.1002/advs.202308687)
Supplement: Supplementary file 1 — Supporting Information [file ADVS-11-2308687-s001.pdf]

ADVANCED SCIENCE
Open Access

Supporting Information

for *Adv. Sci.*, DOI 10.1002/advs.202308687

Visible Meta-Displays for Anti-Counterfeiting with Printable Dielectric Metasurfaces

Jintao Gong, Lingxing Xiong, Mingbo Pu, Xiong Li, Xiaoliang Ma and Xiangang Luo**

Supporting Information

Visible Meta-displays for Anti-counterfeiting with Printable Dielectric Metasurfaces

Jintao Gong, Lingxing Xiong, Mingbo Pu, Xiong Li, Xiaoliang Ma, and Xiangang Luo**

J. Gong, L. Xiong, M. Pu, X. Li, X. Ma, X. Luo
National Key Laboratory of Optical Field Manipulation Science and Technology, Chinese Academy of Sciences, Chengdu 610209, China.
E-mail: pmb@ioe.ac.cn; lxg@ioe.ac.cn

J. Gong, L. Xiong, M. Pu, X. Li, X. Ma, X. Luo
State Key Laboratory of Optical Technologies on Nano-Fabrication and Micro-Engineering, Institute of Optics and Electronics, Chinese Academy of Sciences, Chengdu 610209, China

M. Pu, X. Li, X. Ma, X. Luo
School of Optoelectronics, University of Chinese Academy of Sciences, Beijing 100049, China

L. Xiong
Key Laboratory for Information Science of Electromagnetic Waves (MoE), Fudan University, Shanghai 200433, China

SI-1. Process flowchart for high-throughput fabrication of meta-displays

In view of the production cost, the second-generation soft mold and the final meta-display replicas are fabricated in batch mode using low-cost high-throughput ultra-violet nanoimprinting lithography (UV-NIL). Specifically, the whole production process can be categorized into three distinct steps: first, the creation of the silicon (Si) master mold, also known as the 1st-generation rigid template; second, the replication of the soft polystamp (PS) mold, referred to as the 2nd-generation soft template; and third, the production of the final meta-display items, as depicted in Figure S1. The Si master mold fabrication procedure for crafting the meta-display duplicates involves the utilization of electron beam writing (Elionix F125) to generate a pattern that is an exact match to the target TPC meta-display. To initiate this process, a layer of photoresist (AR-6200) is applied to the silicon wafer, with an inverse pattern being transferred to the photoresist through electron beam exposure. Following this, a 50 nm thick chromium (Cr) layer is applied to the sample via the electron beam evaporation system (ULVAC ei-5z). Then, a lift-off process is carried out in acetone to define a Cr hard mask on the sample. Subsequently, the sample undergoes etching along the Cr mask using inductively-coupled plasma reactive ion etching (LEUVEN INSTRUMENTS). The Si master mold is considered complete after the removal of any residue from the Cr hard mask, which is achieved by employing a Cr etchant (nitrate wet etching).

In the next phase, liquid photosensitive PS resin is evenly spread over the Si master mold, and a PET substrate is placed on top of the coated PS film. The synthetic soft mold, composed of the PET substrate and the PS structures, is then solidified under ultraviolet light irradiation while maintaining a contact pressure of 5000 Pa. Subsequently, the soft mold and the Si template are carefully separated at a controlled, gradual pace. Finally, the high-throughput UV-NIL manufacturing process for TPC-based meta-display replicas is automatically executed using a commercial NIL equipment (GL8/12 CLIV Gen2, GermanLitho GmbH). The scanning electron microscope (SEM) images and the corresponding inset optical microscope images amplified from the process flowchart depict the first-generation Si rigid template, the second-generation PS soft mold, and the desired meta-display replica (i.e., final product), respectively.

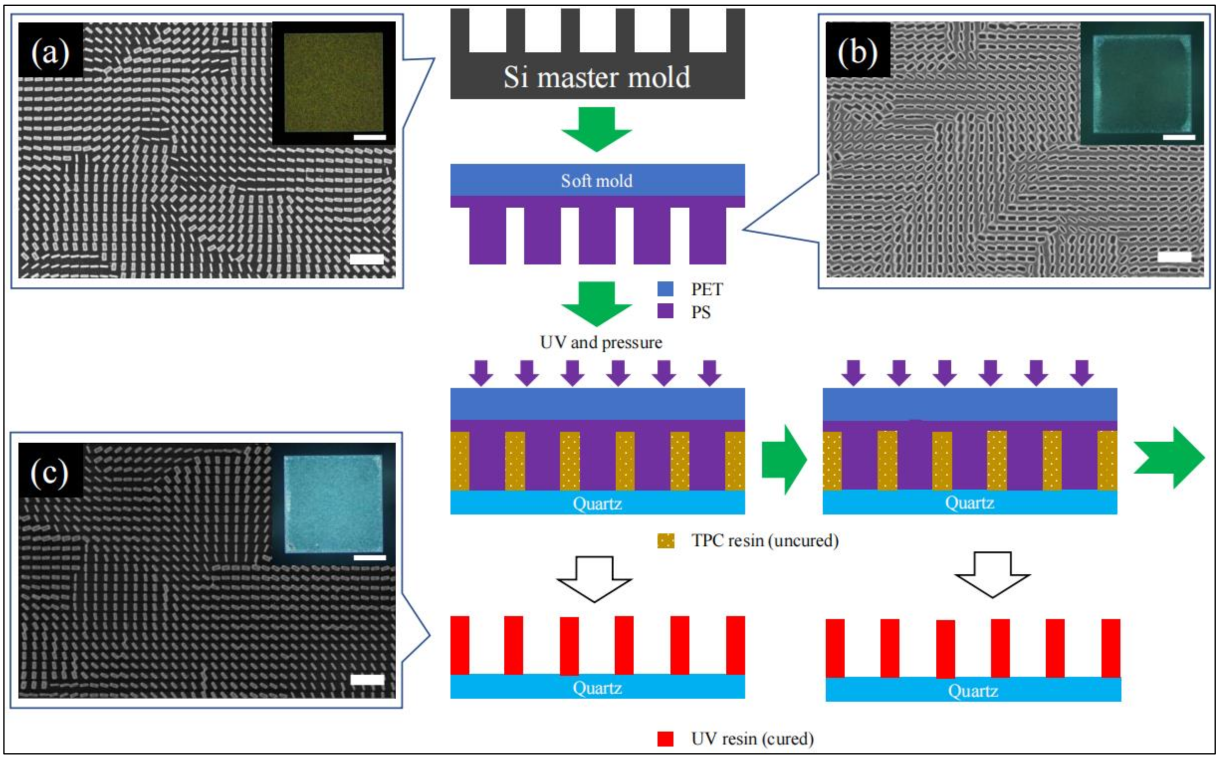

Figure S1. Schematic of the high-throughput fabrication process. a) SEM image of the first-generation rigid template (namely, Si master mold). Inset: the corresponding optical microscope image. b) SEM image of the second-generation flexible template (namely, PS soft mold). Inset: the corresponding optical microscope image. c) SEM image of the final meta-display replica. Inset: the corresponding optical microscope image. The thick white scale bars in SEM images denote a length of 1 μm, and the thin white scale bars in the inset optical microscope images indicate a length of 200 μm.

SI-2. The additional three-channel meta-display sample 3 (MDS3)

To assess the imaging performance of MDS3, our evaluation process commences with the capture of the nanoprinting image of the 1st replica of MDS3, as illustrated in Figure S2(j), utilizing an optical polarizing microscope (Caikon XP-550c). MDS3 is seamlessly integrated into the optical pathway of the microscope for orthogonal polarization, accompanied by a white light source from a halogen lamp. Employing a 10× objective and a micro digital camera (Model: CKC2000), we document the light intensity distribution near the MDS3 surface in transmission mode. The results distinctly reveal an image of Isaac Newton's picture, confirming MDS3's effective performance in amplitude modulation in the near field. The presence of minor noise speckles can be attributed to the near-field interaction between adjacent nanofins, a concern that can be alleviated through the implementation of a more intricate super-cell design. Furthermore, both the 10th replica and the 20th replica exhibit similar near-field nanoprinting imaging behavior, as depicted in Figure S2(k, l). It is worth noting that the experimental observations for all three replicas (replica 1, replica 10, and replica 20) are consistent with the simulation results for near-field greyscale nanoprinting, as presented in Figure S2(i). Subsequently, we employ an alternative optical system to observe the resulting far-field holographic images of these three replicas (replica 1, replica 10, and replica 20), showcased in Figure S2(b-d, f-h). For illuminating MDS3, we employ a tunable super-continuum laser source (NKT-SuperK EXTREME) operating at a specific wavelength of 532 nm. To control the polarization state of the incident beam and achieve circular polarization, we utilize a polarizer and a quarter waveplate (QWP). By rotating the QWP by 90°, we can switch between two independent information channels, leading to the appearance of two distinct far-field holographic images with different QWP configurations.

As presented in Figure S2, the numerous replicas (in this work: replica 1, replica 10, and replica 20) of MDS3 exhibit near-field nanoprinting images that are exceedingly similar and nearly indistinguishable from each other. Moreover, the far-field holographic images they produce are almost identical as well. Notably, when we compare the near-field nanoprinting images of the Isaac Newton portrait on the MDS3 replicas to those of the Niels Bohr portrait, they demonstrate nearly identical grayscale imaging performance. However, it is important to acknowledge that due to the spatial separation of the designed holographic images and the restricted selection of eight unit cell structures (i.e., meta-atoms) for independent control of left-circularly polarized (LCP) and right-circularly polarized (RCP) light incidence, our experiments reveal that when LCP light is incident, the resulting holographic image includes

some slight noise from the RCP-generated holographic image, and vice versa. This spatial separation of holographic images, with the LCP pattern occupying one half and the RCP pattern occupying the other half, implies that half of the meta-atoms for phase control are unavailable, leading to the introduction of unwanted noise from the ineffective half of the unit structures. It is worth noting that these experimental results are consistent with the outcomes of simulations based on vector diffraction theory, as the accompanying holographic pattern (i.e., noise) stemming from the opposite circular polarization can be theoretically predicted. Additionally, it is important to mention that the holographic images we generate are visually clear to the naked eye and enlarge in size as the observation distance increases (i.e., the holographic images diverge). To capture these images, we utilize a white paper as a screen positioned 30 cm away from the sample, and then take photographs using an ordinary smartphone commonly used in daily life. In general, the recorded LCP and RCP holograms closely correspond to the simulated LCP and RCP holographic images, as depicted in Figure S2(a) and S2(e), respectively.

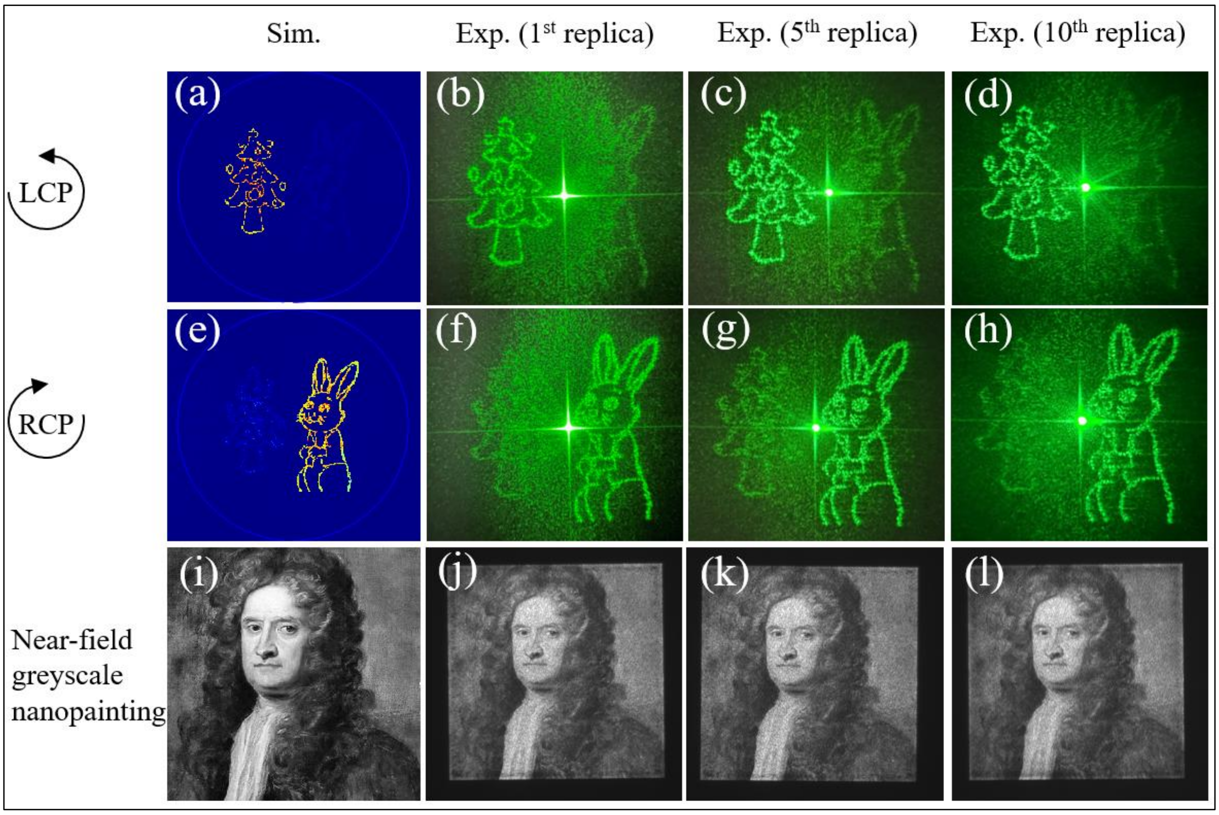

Figure S2. Results of three-channel meta-display sample 3 (MSD3). a) Simulated far-field holographic image (the target image is a sketch of Christmas tree) under LCP light incidence. b-d) Experimentally observed far-field holographic images of the 1st, the 10th, and the 20th replicas, respectively, under LCP light incidence. e) Simulated far-field holographic image (the target image is a sketch of Peter rabbit) under RCP light incidence. f-h) Experimentally observed far-field holographic images of the 1st, the 10th, and the 20th replicas, respectively, under RCP light incidence. i) Simulated near-field greyscale nanoprinting image (the target image is a portrait of Isaac Newton) when inserted in an orthogonal-polarization optical pathway. b-d) Experimentally observed greyscale nanoprinting images of the 1st, the 10th, and the 20th replicas, respectively, when inserted in an orthogonal-polarization optical pathway. Note: The portrait of Isaac Newton is cropped from Charles Jervas' painting of Isaac Newton (1642-1727).

SI-3. Optical setup for characterizing 2-channel and 3-channel meta-display samples

The experimental setup used to observe the far-field holographic images of the three replicas (replica 1, replica 10, and replica 20) corresponding to the meta-display sample 1 (MDS1) is shown in Figure S3(a). To illuminate MDS1, we utilize a tunable super-continuum laser source (NKT-SuperK EXTREME) with a specific wavelength of 532 nm. In order to manage the polarization state of the incoming beam and achieve circular polarization, we employ both a linear polarizer (LP) and a quarter waveplate (QWP). A 90° rotation of the QWP enables us to alternate between two separate information channels, resulting in the emergence of two discernible far-field holographic images characterized by distinct QWP settings.

To evaluate the imaging capabilities of the massively produced replicas of three-channel meta-display samples (MDS2 and MDS3), our assessment process begins by capturing the near-field nanoprinting image of the first replica of MDS2, as depicted in the middle section of Figure S3(b). As for the experimental observations of far-field holographic images, the corresponding optical system for LCP (RCP) light incidence is shown in the top (bottom) section of Figure S3(b).

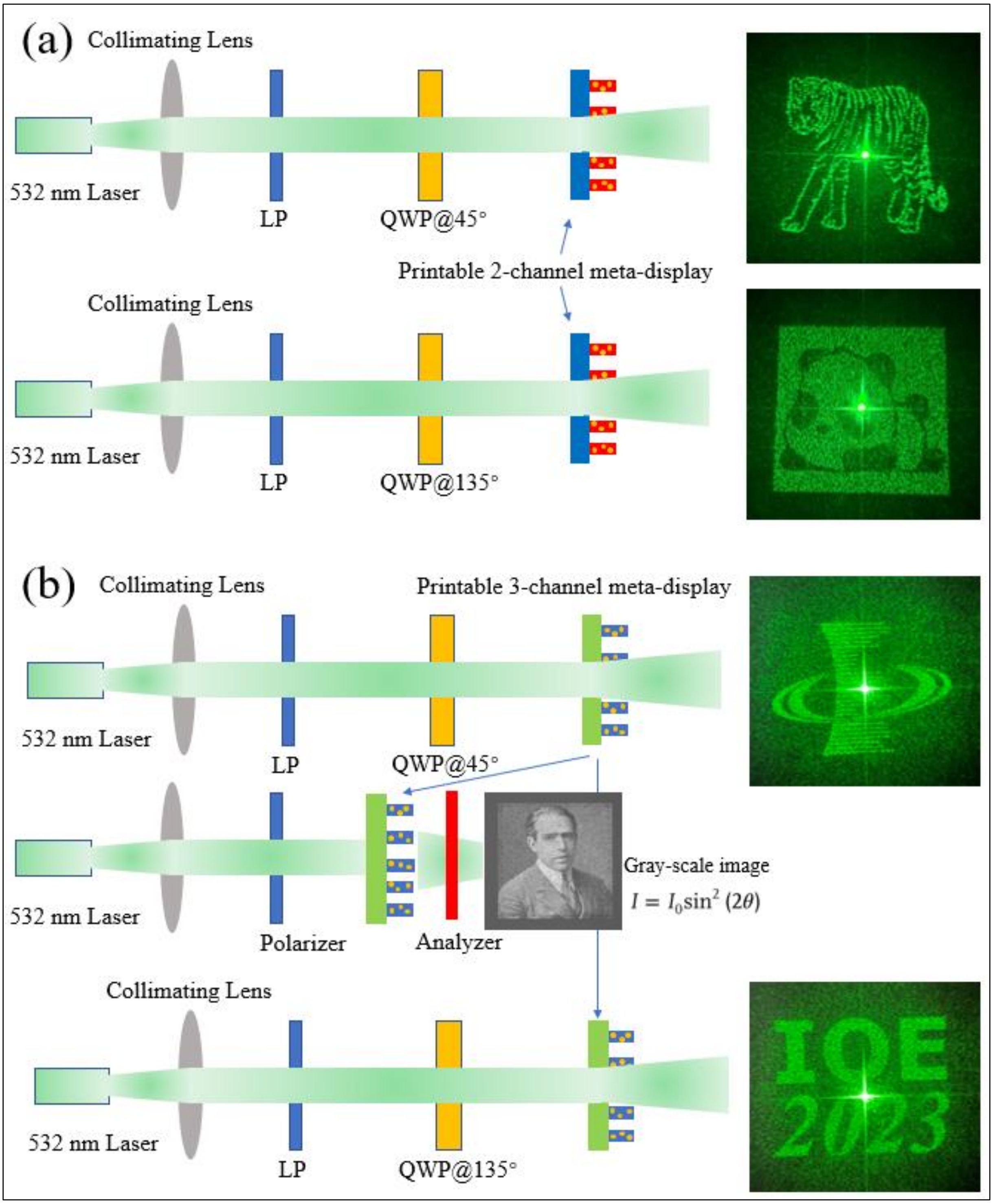

Figure S3. Schematic illustrations of the optical setups for characterizing the scalably manufactured replicas of 2-channel and 3-channel meta-display samples. a) Schematic diagram of the experimental setup for evaluating the imaging capabilities of replicas from the two-channel meta-display sample 1 (MDS1). b) Schematic diagram of the optical system for evaluating the imaging capabilities of replicas from the three-channel meta-display samples (MDS2 and MDS3).

SI-4. The operational principle for achieving high-efficiency broadband performance

To scrutinize the underlying mechanism responsible for the high-efficiency broadband performance of meta-display samples, we conduct simulations to evaluate the amplitude (i.e., polarization conversion ratio, PCR) and propagation phase for incident light at various wavelengths, specifically 473 nm (Blue), 532 nm (Green), and 633 nm (Red). The corresponding results can be observed in Figure S4. Notably, the particular data values of PCR and propagation phase for 473 nm (Blue), 532 nm (Green), and 633 nm (Red) wavelengths are displayed in Table S1, Table S2, and Table S3, respectively.

It is evident that the PCR remains consistently above 0.6, although it diminishes as the operating wavelength deviates from the designated 532 nm in our work. Regarding the propagation phase, specifically the phase component of the cross-polarization, it continues to span the complete range from 0 to 2π when the illuminating light's wavelength deviates from the designated 532 nm. In our holographic image simulations, we consider not only the propagation phase but also the geometric phase (wavelength-independent), as well as the amplitude of the cross-polarization conversion (i.e., PCR). As shown in Figure S4(a), due to the fact that green light (532 nm) has the highest PCR, followed by blue light (473 nm) and then red light (633 nm), we can predict that designed green-light holographic images will be the brightest, blue-light holographic images will be somewhat less bright, and red-light holographic images will be the darkest. This predication can also be experimentally verified as observed from Figure 6 in the main text. Generally, Figure 6 depicts the broadband response measurement results, demonstrating that holographic images for both red light and blue light exhibit decent fidelity. Finally, it is worth noting that the PCR fluctuation among the eight meta-atoms becomes more pronounced as the wavelength deviation increases, potentially leading to increased noise in the near-field nanoprinting image and far-field holographic images.

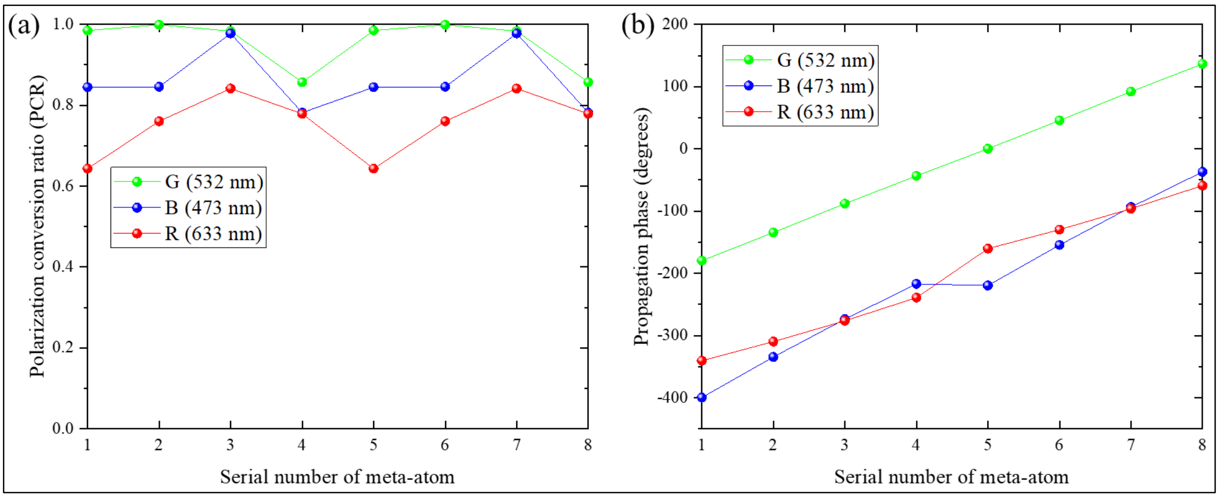

Figure S4. Simulation results of PCR and propagation phase for R (633 nm), G (532 nm), and B (473 nm) light illuminations. a) Simulated PCR results of eight meta-atoms for RGB light illuminations. b) Simulated propagation phase results of eight meta-atoms under the incidence of RGB light sources.

Table S1. Simulation results of PCR and propagation phase for 633 nm light incidence

Unit	#1	#2	#3	#4	#5	#6	#7	#8
Lx	400	405	410	410	90	110	130	155
Ly	90	110	130	155	400	405	410	410
Phase (°)	-340.4	-309.7	-276.2	-239.0	-160.4	-129.7	-96.2	-59.0
PCR	0.6435	0.7607	0.8416	0.7790	0.6435	0.7607	0.8416	0.7790

Table S2. Simulation results of PCR and propagation phase for 532 nm light incidence

Unit	#1	#2	#3	#4	#5	#6	#7	#8
Lx	400	405	410	410	90	110	130	155
Ly	90	110	130	155	400	405	410	410
Phase (°)	-179.7	-134.4	-88.0	-43.4	0.3	45.6	92.0	136.6
PCR	0.9851	0.9991	0.9835	0.8570	0.9851	0.9991	0.9835	0.8570

Table S3. Simulation results of PCR and propagation phase for 473 nm light incidence

Unit	#1	#2	#3	#4	#5	#6	#7	#8
Lx	400	405	410	410	90	110	130	155
Ly	90	110	130	155	400	405	410	410
Phase (°)	-399.6	-334.4	-273.6	-216.9	-219.6	-154.4	-93.6	-36.9
PCR	0.8450	0.8457	0.9776	0.7819	0.8450	0.8457	0.9776	0.7819

SI-5. Photos of the corresponding replicas used in the experimental measurements

As shown in Figure S5, the replication process can be executed using an array-based pattern transfer method, which can save both time and space costs. From the massively produced replicas (replica 1, replica 10, and replica 20), we can clearly see that the replicas perform well at the macro scale, with no visible defects to the naked eye, and each replica looks nearly identical.

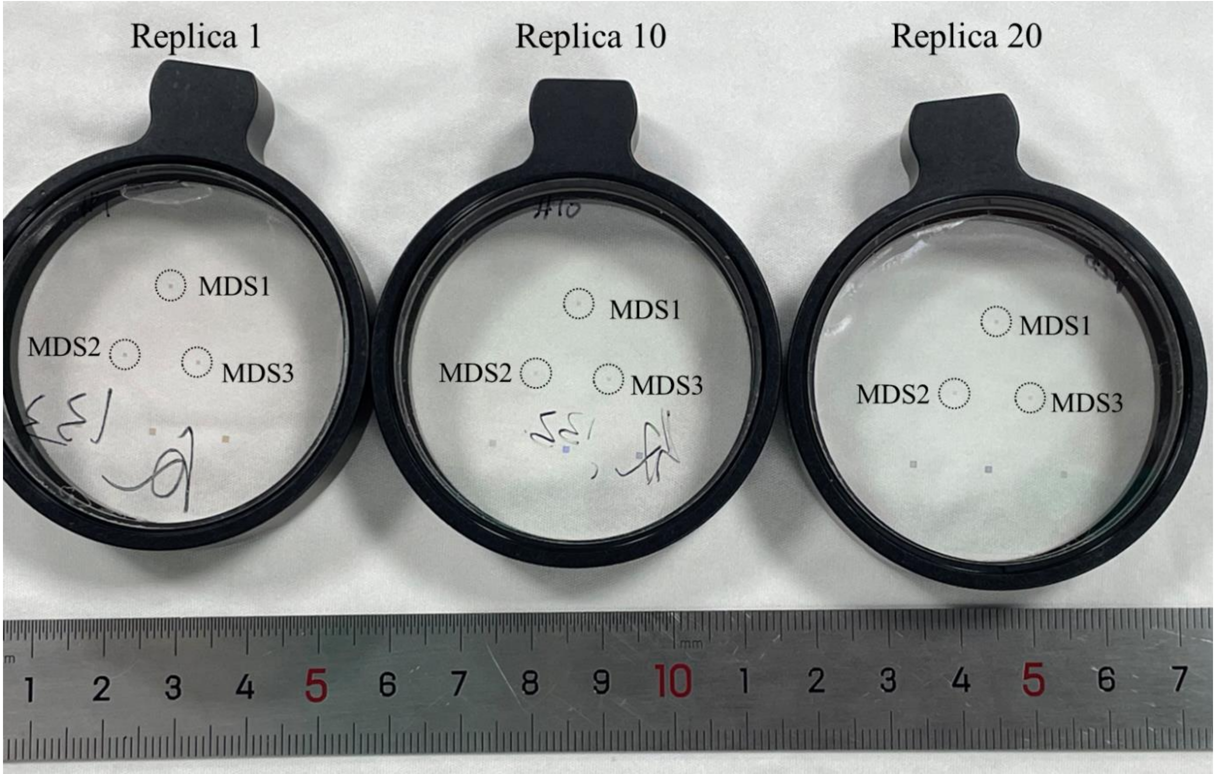

Figure S5. Photos of the 1st replica, the 10th replica, and 20th replica of the designed meta-display samples (MDS1, MDS2 and MDS3). The MDS1 pattern is situated at the top, MDS2 pattern is located on the left side of the middle layer, and MDS3 pattern is located on the right side of the middle layer. Note: The three remaining larger patterns in the bottom layer are designed and fabricated for other optical testing purposes.

SI-6. Analysis of the influence of produced 3D trapezoidal TPC meta-atoms

From the inset of Fig. 3(a) in the main text, it can be clearly observed that the 1st-generation Si template features a 3D trapezoidal shape. Therefore, it is unavoidable that the final TPC replicas feature the same/similar 3D trapezoidal shape. It is important to recognize that this phenomenon is a common occurrence stemming from the silicon master template. To fully address this issue, continuous progress and development in silicon high aspect ratio etching processes are needed from both industry and academia. For the time being, to assess the impact of these massively produced 3D trapezoidal TPC meta-atoms, we conduct corresponding FDTD simulations (Figure S6) and confirm that their influence on the final holographic imaging is minimal (Table S4). Specifically speaking, compared to Table S2, on the one hand, the discretized phase of 3D trapezoidal meta-atoms is relatively uniform, essentially with a phase difference of $\pi/4$. On the other hand, 3D trapezoidal meta-atoms also exhibit a fairly high average PCR value of 91.2%. This is comparable to the simulated PCR of 3D rectangular meta-atoms, which has an average value of 95.6%. Overall, the shape errors resulting from the fabrication process have a negligible impact on the final meta-display performance.

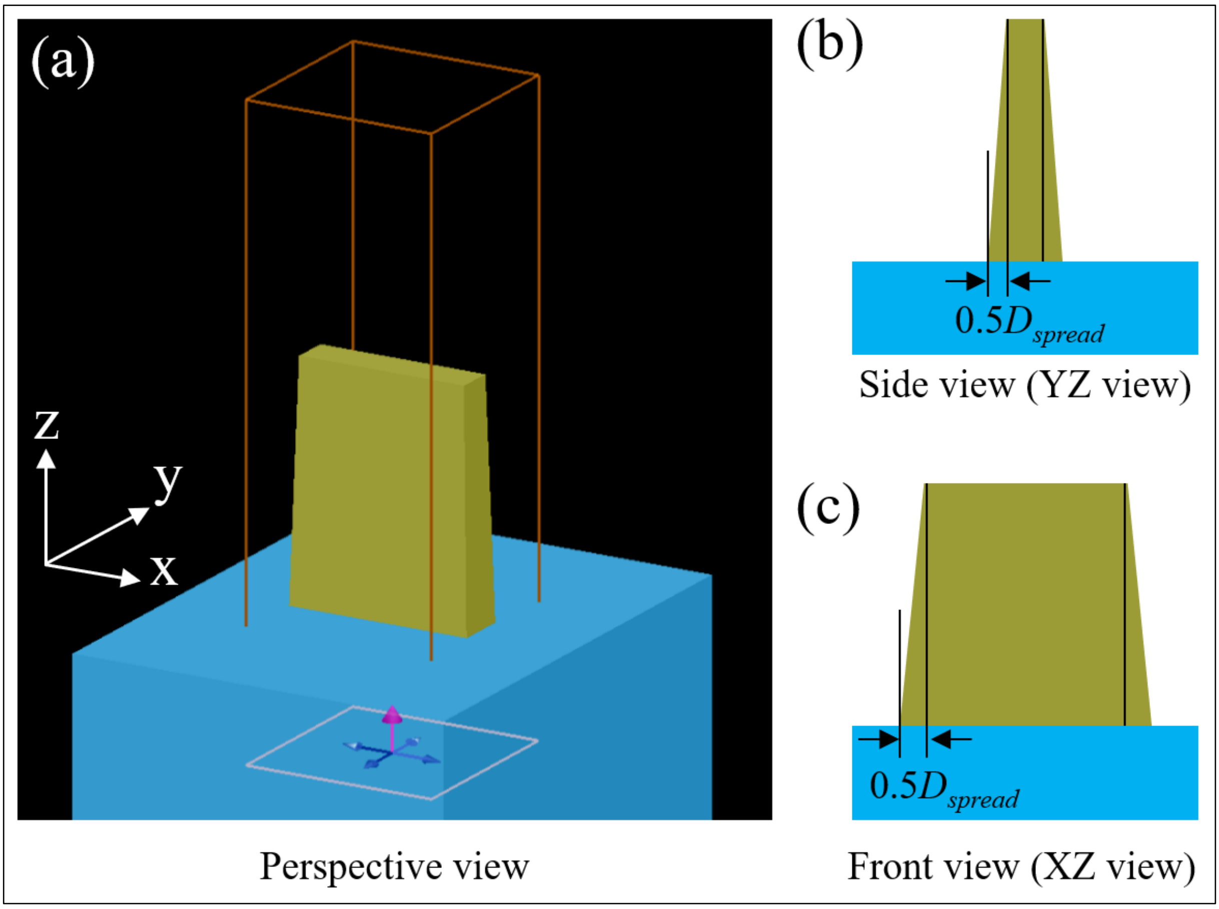

Figure S6. Simulation of produced 3D trapezoidal TPC meta-atoms. a) Perspective view of the established 3D trapezoidal TPC meta-atom model in the FDTD simulation. b) Side view of the modeled TPC meta-atom. c) Front view of the modeled TPC meta-atom. Note: D_{spread} is a structural parameter to indicate the lateral spread of the bottom structure of the 3D trapezoidal shape. Based on SEM characterization results in the main text, D_{spread} is set as 20 nm.

Table S4. Simulation results of PCR and propagation phase for 532 nm light incidence (3D trapezoidal shape with D_{spread} = 20 nm)

Unit	#1	#2	#3	#4	#5	#6	#7	#8
Lx	400	405	410	410	90	110	130	155
Ly	90	110	130	155	400	405	410	410
Phase (°)	-142.6	-96.4	-49.0	-8.9	37.4	83.6	131.0	171.1
PCR	0.9984	0.9995	0.8931	0.7682	0.9984	0.9995	0.8931	0.7682

SI-7. Elimination of unmodulated light via an additional optical filtering system

In fact, the elimination of holographic image noise can be achieved effectively. As depicted in Figure S7, a viable approach involves filtering the unmodulated co-polarization light through an additional optical filtering system (QWP+LP), resulting in a distinct holographic image. A convex lens is employed to reduce the filtered clear holographic image for capture by a CMOS camera (Prosilica GT2050NIR, Allied Vision). It is noteworthy that the edge distortion (barrel distortion) in the full-frame holographic image (e.g., cartoon Panda, characters “IOE 2023”) is attributed to the imaging characteristics of the convex lens and the presence of a specific flange distance between the convex lens and the CMOS camera.

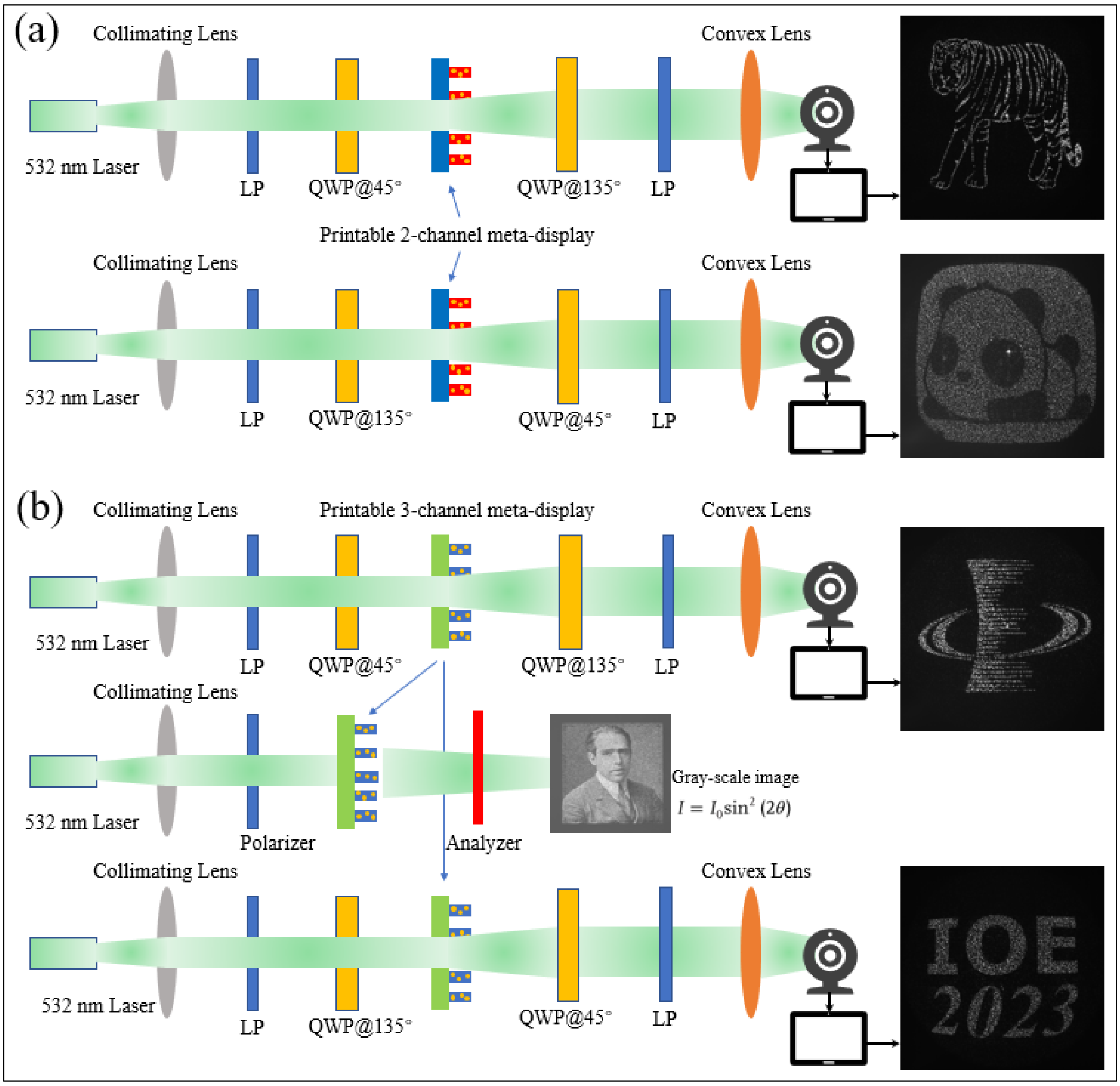

Figure S7. Schematic illustrations of the optical setups for obtaining distinct holographic images. a) Schematic diagram of the experimental setup for characterizing replicas from the two-channel meta-display sample 1 (MDS1). b) Schematic diagram of the optical system for characterizing replicas from the three-channel meta-display samples (MDS2 and MDS3).

SI-8. PCR efficiency measurements for mass-produced meta-display replicas

To fully cover the sample, the input beam's diameter is set to 1000 μm with the aid of a pinhole. To determine the power of the input beam, an initial measurement is taken at the position between the pinhole and the meta-display sample, as depicted in Figure S8(b). Additionally, it should be noted that the sample has dimensions of 540 μm × 540 μm, as illustrated in Figure S8(c). Therefore, the power distributed on the sample constitutes a proportion, denoted as k, given by

$$k = \frac{4 \times 0.54^2}{\pi} = 37.13\%$$

It is important to highlight that the calculation of the equivalent power for sample is on this basis. To assess the polarization conversion ratio (PCR) in the experiment, a co-polarized analyzer (QWP+LP) is utilized, selectively blocking the unmodulated beam (co-polarized beam) and permitting only the converted beam (cross-polarized beam) to pass through. The power of the focused converted beam is then measured using a convex lens and an optical power meter (1919-R, Newport), as illustrated in Figure S8(a). Due to the primary focus of this study on the batch fabrication of visible meta-displays, we conducted PCR measurements on the first, tenth, and twentieth replicas of MDS1. The incident light for these measurements was right-handed polarized, corresponding to a holographic pattern depicting a cartoon Panda. The corresponding measurement results are summarized in Table S5.

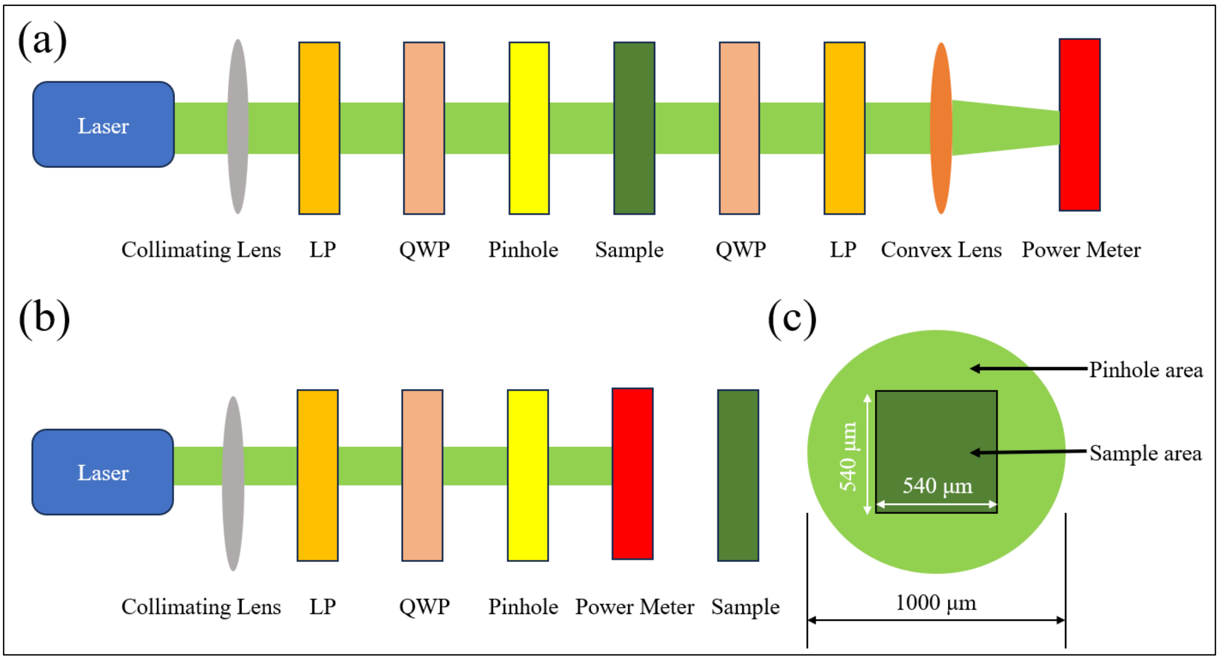

Figure S8. Schematics for PCR measurements. a) Schematic diagram of the experimental setup for measuring the cross-polarized beam after the optical filter system. b) Schematic diagram of the optical setup for measuring the input beam after the pinhole. c) Size of the pinhole and size of the meta-display sample.

Table S5. Measurement results of PCR efficiency for 1st, 10th, 20th replicas from MDS1 under RCP light incidence

	Measured power after 1 mm pinhole (μW)	Equivalent power for sample (μW)	Measured power after optical filter system (μW)	Calculated efficiency
Replica 1	**4.34**	1.61	**1.46**	90.7%
Replica 10	**4.38**	1.63	**1.44**	88.3%
Replica 20	**4.25**	1.58	**1.41**	89.2%
Remarks / Average Efficiency	See Fig. S8(b) for the measurement setup	Refer to Fig. S8(c) for the conversion basis	See Fig. S8(a) for the measurement setup	**89.4%**
